# Displacement of Slow-Turnover DNA Glycosylases by Molecular Traffic on DNA

**DOI:** 10.3390/genes11080866

**Published:** 2020-07-30

**Authors:** Anna V. Yudkina, Anton V. Endutkin, Eugenia A. Diatlova, Nina A. Moor, Ivan P. Vokhtantsev, Inga R. Grin, Dmitry O. Zharkov

**Affiliations:** 1 Siberian Branch of the Russian Acasemy of Sciences Institute of Chemical Biology and Fundamental Medicine, 8 Lavrentieva Ave., 630090 Novosibirsk, Russia; ayudkina@niboch.nsc.ru (A.V.Y.); aend@niboch.nsc.ru (A.V.E.); e.diatlova@g.nsu.ru (E.A.D.); moor@niboch.nsc.ru (N.A.M.); i.vokhtantsev@g.nsu.ru (I.P.V.); grin@niboch.nsc.ru (I.R.G.); 2 Novosibirsk State University, 2 Pirogova St., 630090 Novosibirsk, Russia

**Keywords:** DNA damage, DNA repair, tight protein–DNA complexes, DNA polymerases, facilitated diffusion, molecular traffic

## Abstract

In the base excision repair pathway, the initiating enzymes, DNA glycosylases, remove damaged bases and form long-living complexes with the abasic DNA product, but can be displaced by AP endonucleases. However, many nuclear proteins can move along DNA, either actively (such as DNA or RNA polymerases) or by passive one-dimensional diffusion. In most cases, it is not clear whether this movement is disturbed by other bound proteins or how collisions with moving proteins affect the bound proteins, including DNA glycosylases. We have used a two-substrate system to study the displacement of human OGG1 and NEIL1 DNA glycosylases by DNA polymerases in both elongation and diffusion mode and by D4, a passively diffusing subunit of a viral DNA polymerase. The OGG1–DNA product complex was disrupted by DNA polymerase β (POLβ) in both elongation and diffusion mode, Klenow fragment (KF) in the elongation mode and by D4. NEIL1, which has a shorter half-life on DNA, was displaced more efficiently. Hence, both possibly specific interactions with POLβ and nonspecific collisions (KF, D4) can displace DNA glycosylases from DNA. The protein movement along DNA was blocked by very tightly bound Cas9 RNA-targeted nuclease, providing an upper limit on the efficiency of obstacle clearance.

## 1. Introduction

Genomic DNA is bound to a number of structural, regulatory and catalytic proteins, some of which form very strong non-covalent complexes with DNA. In mammalian cells, the most abundant of these proteins are chromatin structural components involved in DNA packaging and nuclear architecture (histones, condensins, cohesins, high-mobility group proteins, etc.) [1,2,3]. However, there is also a variety of nonstructural proteins tightly bound to DNA. Inevitably, site-specific and site-nonspecific proteins compete for binding DNA targets. Transcription and replication elongation complexes unwind the helix and proceed actively displacing some protein obstacles but may be blocked by others [4]. Moreover, many proteins have been shown to move along DNA non-directionally by facilitated diffusion [5,6], and it is generally not known how they move over roadblocks presented by tightly bound molecules of other proteins. The competition between proteins stably bound to DNA and moving along it is sometimes referred to as “molecular traffic jam” on DNA [4]. Given the diversity of pathways in which DNA-bound proteins participate, most of collisions on DNA are expected to occur between functionally unrelated proteins and the outcome of such collisions is probably guided by thermodynamics rather than by specific protein–protein interactions.

Base excision DNA repair (BER) is an important process that counteracts the ever-present threat of DNA damage. In BER, damaged DNA is restored to normal by the sequential action of several enzymes—DNA glycosylases, AP endonucleases, DNA polymerases and DNA ligases [7,8]. Many DNA glycosylases, the enzymes that initiate BER through excision of damaged bases, have very high affinity for the reaction product, the apurinic/apyrimidinic (AP) site, and therefore have a slow turnover [9,10]. This may be biologically important since AP sites are potentially more dangerous for cells than the original damaged bases. Thus, in many cases, the AP site product is directly handed over from a DNA glycosylase to an AP endonuclease [9,10]. Notably, DNA glycosylases and AP endonucleases do not form stable complexes, and the handover is achieved in a dynamic manner, with AP endonucleases displacing DNA glycosylases from the nascent AP site. The displacement also increases the glycosylase turnover, assisting the flow of substrate through the BER pathway [11,12]. In human BER, the stimulation by the major AP endonuclease APEX1 has been demonstrated so far for 8-oxoguanine–DNA glycosylase OGG1 [12,13,14,15,16], mismatched adenine–DNA glycosylase MUTYH [17,18], uracil–DNA glycosylase UNG [19], thymine–DNA glycosylase TDG [20,21], single-strand-specific monofunctional uracil–DNA glycosylase SMUG1 [22,23], methylpurine–DNA glycosylase MPG [24,25] and endonuclease III-like protein NTHL1 [26]. Several examples of stimulation of DNA glycosylases by proteins other than AP endonucleases have been reported (reviewed in [10]). However, it is unclear whether these interactions are specific or any protein moving along DNA in an energy-dependent or-independent manner could also displace DNA glycosylases.

Here we address the question whether DNA glycosylases can be displaced by other proteins moving along DNA in the active elongation mode or passive diffusion mode. We address the displacement of a slow-turnover 8-oxoguanine–DNA glycosylase (OGG1) by DNA polymerase β, which may be specific since both proteins belong to the human BER system and by the Klenow fragment of *Escherichia coli* DNA polymerase I (KF) and vaccinia virus D4 protein, used here as models of nonspecific DNA-directed interactions. Further, we confirm these results using another DNA glycosylase, NEIL1, which is known to be stimulated by several proteins other than APEX1. Finally, we assess the ability of proteins moving along DNA to remove very tightly bound obstacles using an extremely slow-turnover Cas9 RNA-targeted nuclease as a model.

## 2. Materials and Methods

### 2.1. Oligonucleotides and Enzymes

Phage T7 RNA polymerase was from Biosan (Novosibirsk, Russia) and *E. coli* Ung, from SibEnzyme (Novosibirsk, Russia). Exonuclease-deficient Klenow fragment of *E. coli* DNA polymerase I (KF), rat DNA polymerase β (POLβ), bacteriophage RB69 DNA polymerase, human OGG1 and NEIL1 DNA glycosylases, vaccinia virus D4 protein, *Streptococcus pyogenes* nuclease Cas9 and its catalytically inactive form dCas9 (Cas9 D10A H840A) were overexpressed and purified essentially as described [27,28,29,30,31,32,33]. Single-guide RNA for Cas9 and dCas9 was synthesized by in vitro transcription with T7 RNA polymerase and gel-purified according to a published protocol [34].

**Figure 1:**
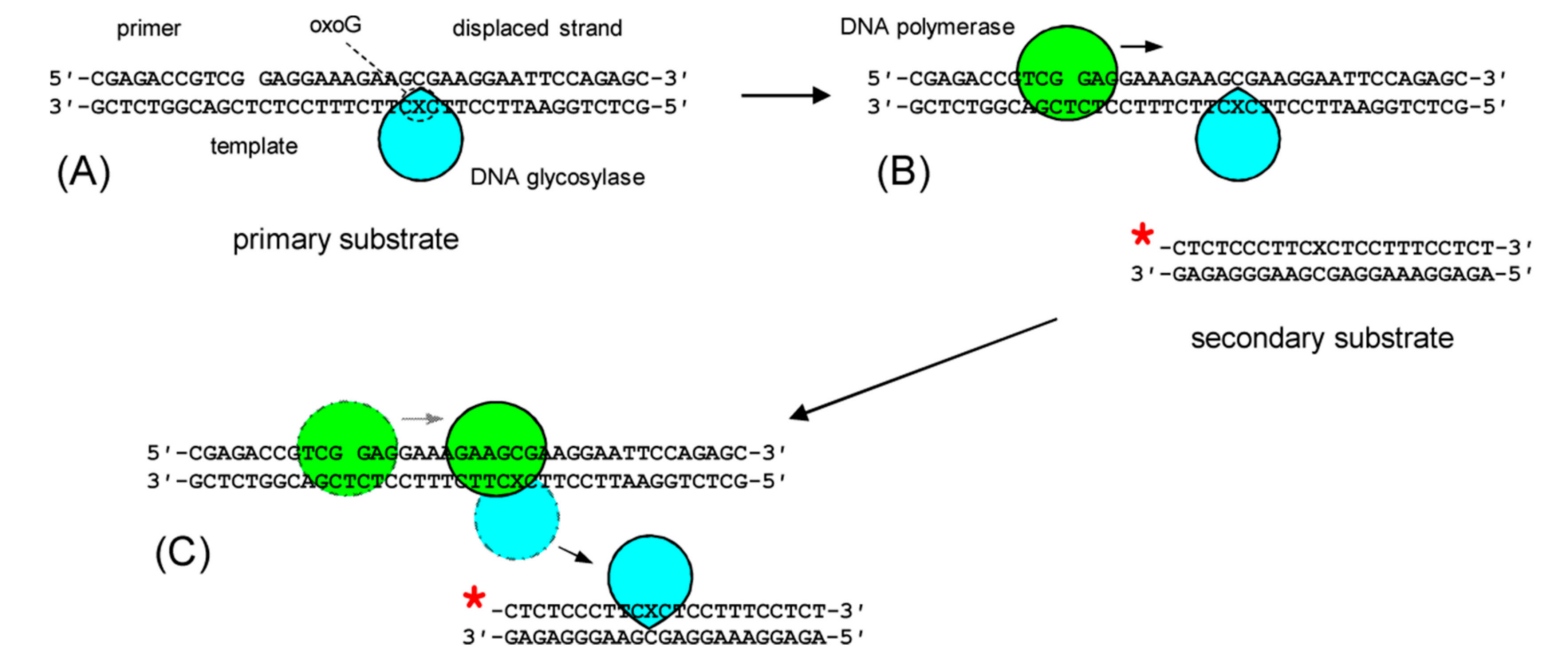
General scheme of a two-substrate experiment to measure rate enhancement of slow-turnover enzymes by proteins moving along DNA. (**A**) DNA glycosylase (OGG1 or NEIL1 in our experiments) is bound to the unlabeled primary substrate; in the absence of other enzymes it turns over slowly; (**B**) DNA polymerase (or other protein of interest) and the labeled secondary substrate is added; (**C**) DNA polymerase displaces the DNA glycosylase, which binds and cleaves the secondary substrate. Red asterisk denotes the radioactive label at the 5′-terminus.

Oligonucleotides (Table 1) were synthesized in-house from commercially available phosphoramidites (Glen Research, Sterling, VA, USA) and purified by reverse-phase HPLC on a PRP-1 C_18_ column (Hamilton, Reno, NV, USA). If necessary, oligonucleotides were 5′-labeled using γ[^32^P]-ATP (Institute of Chemical Biology and Fundamental Medicine Laboratory of Biotechnology, Novosibirsk, Russia) and phage T4 polynucleotide kinase (Biosan) according to the manufacturer’s protocol. The scheme of substrate assembly is shown in Figure 1. To prepare AP site-containing substrates, 1-μM oligonucleotide duplexes with a dU residue were treated with 5 U of Ung for 15 min at 37 °C in a buffer containing Tris–HCl (pH 8.0), 1-mM EDTA and 1-mM DTT, desalted on Sephadex G-25 and immediately used in the reaction.

### 2.2. OGG1 Displacement From Its Complex with DNA

The binding mixture contained 50-mM Tris–HCl (pH 7.5), 10-mM NaCl, 0.1-mM EDTA, 0.1-mM DTT, 0.5-mM each dNTP, 10-nM OGG1 and 20-nM non-labeled gapped oxoG substrate (P–DX//TC or P–DC//TX; primary substrate). The mixture was incubated for 2 min at 37 °C to allow complete binding of OGG1 to the non-labeled substrate. To follow the release of OGG1 in presence of displacing enzymes, the mixture was supplemented with 100-nM ^32^P-labeled secondary substrate (*S2X//2S) and 10-nM enzyme of choice (20-nM for D4 protein) in the appropriate buffer: 10-mM Tris–HCl (pH 7.6), 10-mM MgCl_2_ and 1-mM DTT for Pol β; 50-mM Tris–HCl (pH 7.5), 5-mM MgCl_2_, 1-mM EDTA, 30-mM KCl and 5-mM DTT for KF; 20-mM Tris–HCl (pH 8.0), 1-mM EDTA and 1-mM DTT for D4 protein, or with the buffer alone. The final volume of the reaction mixture was 30 µL. The reaction was allowed to proceed for 2–20 min at 37 °C. At the required time, 5-µL aliquots were withdrawn, quenched with 0.1-M NaOH, heated for 2 min at 95 °C, neutralized with the equimolar amount of HCl and mixed with an equal volume of formamide dye solution (80% formamide, 20-mM Na-EDTA, 0.1% xylene cyanol, 0.1% bromophenol blue). The products were resolved by 20% denaturing PAGE, visualized and quantified using by phosphorimaging (Typhoon FLA 9500, GE Healthcare, Chicago, IL, USA).

### 2.3. Decay of OGG1–DNA Covalent Intermediates

The conditions for OGG1 binding and polymerase reaction initiation were identical to those described above except that no secondary substrate was added and the oxoG-containing strand in the primary substrate was ^32^P-labeled. The concentrations of OGG1 and the primary substrate were 50 nM. At the required time (2–40 min), 5-µL aliquots were withdrawn, mixed with 5 µL of freshly prepared 0.2-M NaBH_4_ solution, and incubated for 30 min at 37 °C. The products were resolved by 12% discontinuous PAGE (Lemmli system), visualized and quantified using by phosphorimaging. The decay curves were fit by an exponential decay model using SigmaPlot v11 (Systat Software, San Jose, CA, USA).

### 2.4. NEIL1 Displacement from Its Complex with DNA

The reaction was identical to that described above for OGG1 except the binding mixture contained 20-nM NEIL1 and 20-nM non-labeled gapped AP substrate (P–DU//TC or P–DC//TU after Ung treatment) and the secondary substrate contained 5-hydroxyuracil instead of oxoG (*S2Y//2S).

### 2.5. Cas9 Displacement from Its Complex with DNA

Immediately before the reaction, Cas9 or dCas9 complexes with sgRNA were reconstituted by incubation of 2.5-μM protein and 2.5-μM sgRNA for 15 min at 37 °C in a buffer containing 10-mM HEPES–KOH (pH 7.5), 250-mM KCl, 0.5-mM DTT and 25% glycerol. The binding mixture contained 10-mM HEPES–KOH (pH 7.5), 50-mM KCl, 0.25-mM EDTA, 0.5-mM DTT, 2.5% glycerol, 0.5-mM each dNTP, 80-nM Cas9 or dCas9 and 100-nM non-labeled gapped Cas9 primary substrate (P–DCas//TCas). The mixture was incubated for 2 min at 37 °C to allow Cas9-binding. To follow the release of Cas9, the mixture was supplemented with 100-nM ^32^P-labeled secondary substrate (*S2Cas//Cas2S) and 0-nM or 100-nM enzyme of choice (DNA polymerase or D4 protein) and treated as described above for OGG1. For RB69 DNA polymerase, the reaction buffer was 50-mM Tris–HCl (pH 7.5), 5-mM MgCl_2_ and 0.1-mM DTT.

### 2.6. Primer Elongation in the Presence of Protein Obstacles

The binding mixtures were identical to those described above except that the primer strand in the primary substrate was ^32^P-labeled, and, in the case of Cas9, 1-μM dCas9 substituted for Cas9. The mixture was incubated for 2 min at 37 °C to allow protein-binding, supplemented with DNA polymerases without the secondary substrate and treated as described above for OGG1.

### 2.7. Microscale Thermophoresis

His_6_-tagged OGG1 was labeled with red Tris-NTA dye and non-tagged POLβ, with cysteine-reactive green maleimide dye (NanoTemper Technologies, Munich, Germany), and purified according to manufacturer’s protocol. All reaction mixtures with a final volume of 10 μL consisted of labeled protein (30 nM), varying amounts of the unlabeled binding partner, 100 mM Tris-HCl (pH 7.5), 10 mM MgCl_2_ and 1% (*v/v*) glycerol. Ligand titrations were performed by serial dilutions. Measurements were carried out using standard capillaries in the Monolith NT.115 device (NanoTemper Technologies) equipped with a red/green detection channel and medium infrared laser power. The data were fitted to a one-site-binding model using SigmaPlot.

### 2.8. DNA-Guided Protein–Protein Docking

A random sequence linear B-DNA structure generated using the fiber module of 3DNA [35] was used as a docking guide. All operations were done in PyMOL (Schrödinger, New York, NY, USA). The structures for docking included crystal structures of OGG1 bound to double-stranded oxoG-containing DNA (PDB ID 1EBM [36]) and POLβ bound to gapped DNA (2FMP [37]) and the molecular dynamics model of KF bound to gapped DNA [38] based on the crystal structure of KF bound to primer/template DNA (4BDP [39]). The duplex part of a DNA polymerase structure including the downstream primer and the complementary part of the template was aligned to one half of the guide and either 5′- or 3′-DNA shoulder of the OGG1 complex was aligned to another half to superimpose the oxoG residue with either the downstream primer or the template strand. The OGG1 complex was then moved along the helical guide by 1-bp steps until its solvation surface touched the surface of the polymerase.

## 3. Results

### 3.1. OGG1 Can Be Displaced by DNA Polymerase β

The displacement and stimulation of human 8-oxoguanine–DNA glycosylase (OGG1) by the major human AP endonuclease (APEX1) is the best-studied example of transient protein–protein interactions in the BER pathway [12,13,14,15,16,40]. In the absence of APEX1, the half-life of the OGG1–product complex is ~17 min [41], whereas APEX1 accelerates the reaction ~10-fold through the increase in the product release rate constant [12,16]. The affinity of OGG1 for the AP site reaction product is ~2–20 nM [13,41]. To address the issue whether OGG1 can be displaced by other proteins, we have designed a two-substrate system (Figure 1). In this setup, OGG1 is first bound to an unlabeled primary substrate, and then the second enzyme, such as DNA polymerase, is added together with the labeled secondary substrate, which is cleaved by the displaced OGG1. Displacement of OGG1 will be evident as an increase in the rate of cleavage of the secondary substrate. The same primary substrate was used previously to characterize the action of several DNA polymerases on this substrate containing a model protein–DNA cross-link [42].

**Figure 2:**
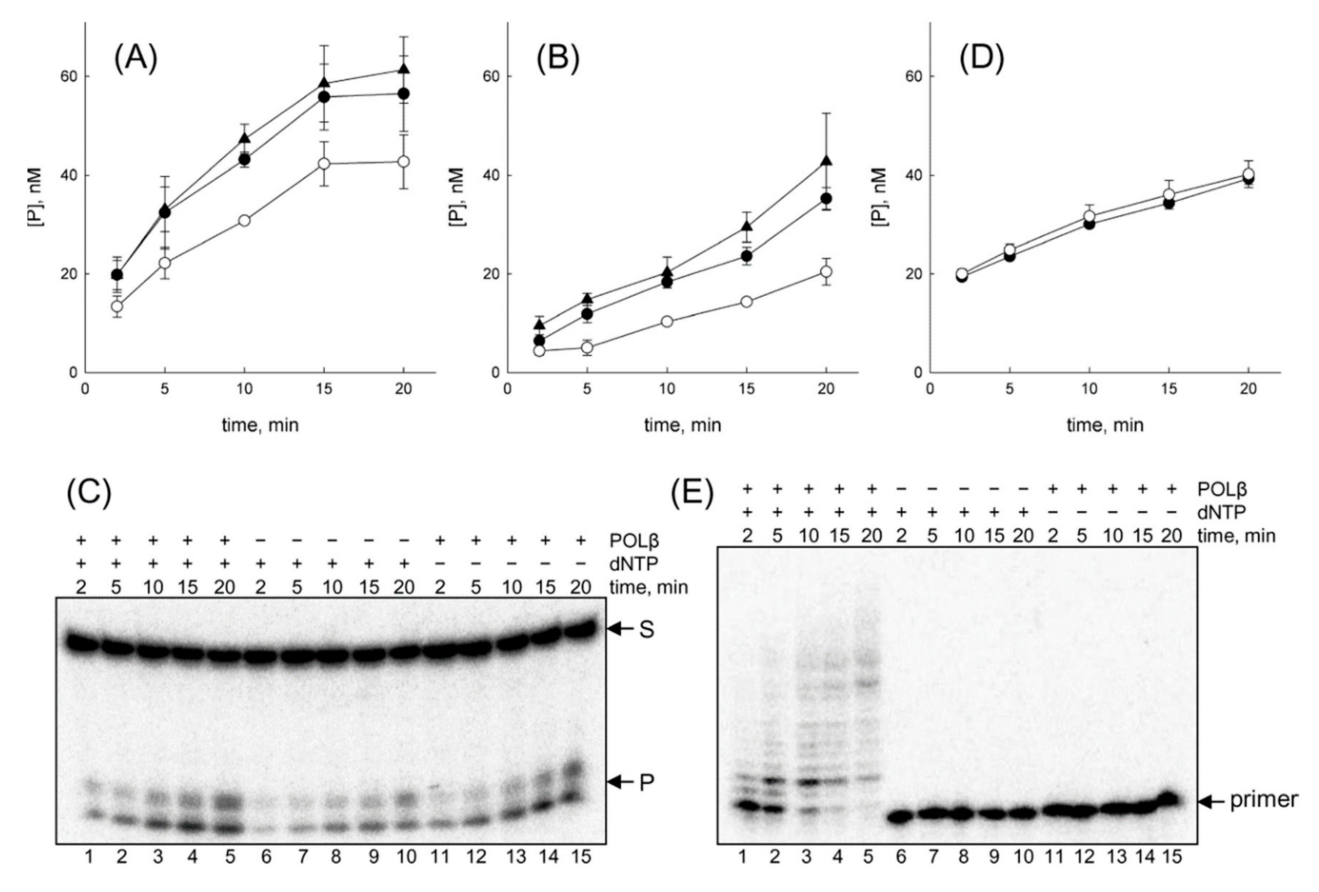
Displacement of 8-oxoguanine–DNA glycosylase (OGG1) by DNA polymerase β. (**A**) POLβ interacting with OGG1 bound to the template strand; (**B**) POLβ interacting with OGG1 bound to the downstream strand. White circles show the secondary substrate cleavage in the absence of POLβ, black circles, in the presence of POLβ and dNTPs and black triangles, in the presence of POLβ without dNTPs. Mean ± SEM of 3–5 independent experiments are presented; (**C**) a representative gel of the cleavage experiment. Lanes 1–5, both POLβ and dNTPs are present; lanes 6–10, no POLβ; lanes 11–15, POLβ without dNTPs. OGG1 was on the downstream strand. The arrows mark the mobility of the substrate (S) and β-elimination product (P). The incubation time is indicated above the gel; (**D**) cleavage of the secondary substrate by OGG1 with or without POLβ in the absence of the primary substrate; (**E**) a representative gel of the primer extension experiment. The substrate and the conditions were the same as in Panel C. The arrow marks the mobility of the primer.

We first inquired whether OGG1 can be displaced by a major mammalian BER enzyme, DNA polymerase β (POLβ). POLβ has relatively low processivity and, if presented with a substrate containing a downstream strand, elongates the primer by 3–5 nucleotides (nt) per association [43,44]; the maximum elongation with strand displacement reported in the literature is ~10 nt [45]. Interestingly, POLβ can be co-immunoprecipitated with OGG1, suggesting possible interaction between these proteins [46]. In our earlier studies with a covalently cross-linked protein obstacle, POLβ was able to extend a DNA primer with partial distortion of the interfering protein globule [42]. A >2-fold enhancement of OGG1 turnover by POLβ was evident both when OGG1 was bound to the template strand and when it was holding the downstream strand (Figure 2A,B). Cleavage of the secondary substrate by OGG1 led to the appearance of a doublet band with higher mobility (Figure 2C), characteristic of β-elimination by DNA glycosylases with the associated AP lyase activity [47,48]. Notably, POLβ was able to displace OGG1 even in the absence of dNTPs (Figure 2A,B), suggesting that these proteins can also interact when POLβ passively diffuses along DNA. No difference was observed in the unstimulated OGG1 cleavage with or without dNTPs (data not shown). In addition, the presence of POLβ without the primary substrate did not change the cleavage of the secondary substrate, a blunt-ended DNA duplex that has low affinity for DNA polymerases (Figure 2D).

To confirm that DNA polymerase reaction occurs in the presence of OGG1, we had employed a primary substrate in which the primer was ^32^P-labeled. The results unambiguously show that POLβ is capable of extending the primer when OGG1 is present (Figure 2E).

**Figure 3:**
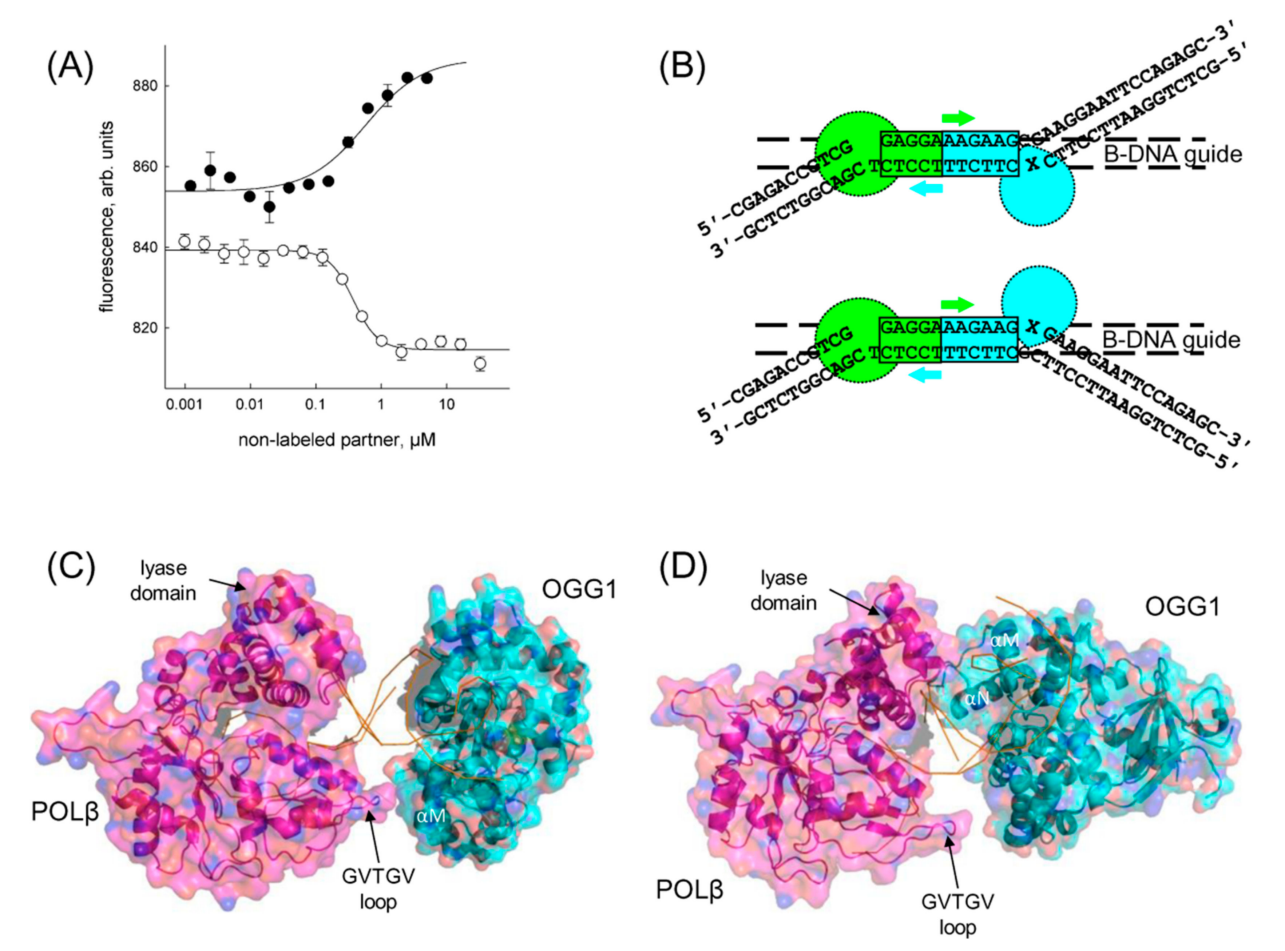
Interactions of OGG1 and DNA polymerase β. (**A**) OGG1–POLβ interaction in solution measured by microscale thermophoresis. White circles, POLβ labeled at Cys residues titrated with unlabeled OGG1; black circles, OGG1 labeled at the His-tag titrated with unlabeled POLβ. Mean ± SEM of three independent experiments are presented; (**B**) scheme of the DNA-guided superposition of OGG1 and POLβ structures (PDB ID 1EBM and 2FMP, respectively). The boxed areas correspond to the DNA parts from the structures superimposed with the simulated B-DNA guide; (**C**,**D**) possible structure of OGG1–POLβ encounter complex predicted by DNA-guided docking; OGG1 is on the downstream (**C**) or the template strand (**D**). OGG1 is colored cyan, POLβ, magenta, DNA is shown as a backbone trace.

To analyze whether OGG1 and POLβ could interact directly in solution, we have estimated the affinity of these proteins for each other using the microscale thermophoresis technology [49]. Introducing a fluorescent moiety into either OGG1 or POLβ and titrating the labeled protein with its unlabeled binding partner (Figure 3A), we obtained the binding affinity values of 580 ± 200-nM and 390 ± 80 nM, respectively, under a single-site-binding model. Given that the concentration of OGG1 and POLβ in the reaction mixture was 10 nM, only a minor fraction of OGG1 would be bound to POLβ in the absence of DNA, and it is likely that dNTP-independent stimulation of OGG1 by POLβ is due to the interaction of the proteins on DNA.

Based on this assumption, we have searched for possible OGG1–POLβ interaction interfaces by DNA-guided protein–protein docking. To do this, we have superimposed the DNA parts of the crystal structures of OGG1–DNA and POLβ–DNA (primer/template/downstream strand) complexes to a simulated B-DNA guide and determined the positions where the protein surfaces come into contact. Since both OGG1 and POLβ kink DNA, only the 5-bp DNA part containing the downstream strand and the nucleotides complementary to it was used to guide the alignment of POLβ, and only the 6-bp part facing the advancing polymerase was used to guide the alignment of OGG1 (Figure 3B). If OGG1 was holding the downstream strand, its αM helix contacted the ^302^GVTGV^306^ loop in the POLβ thumb subdomain (Figure 3C). The αM helix of OGG1 contains the catalytic Asp268 residue [36], so any distortion of αM would presumably terminate the reaction and unfavorably change the conformation of the lesion-binding pocket. If OGG1 was on the template strand, the loop connecting αM and αN helices came into contact with the 8-kDa lyase domain of POLβ (Figure 3D). Here, too, the αM helix may be distorted by the encounter with the polymerase; the αM/αN loop also participated in DNA-binding by OGG1 and thus its deformation can decrease the affinity of OGG1 for DNA.

### 3.2. OGG1 Can Be Nonspecifically Displaced by the Klenow Fragment

**Figure 4:**
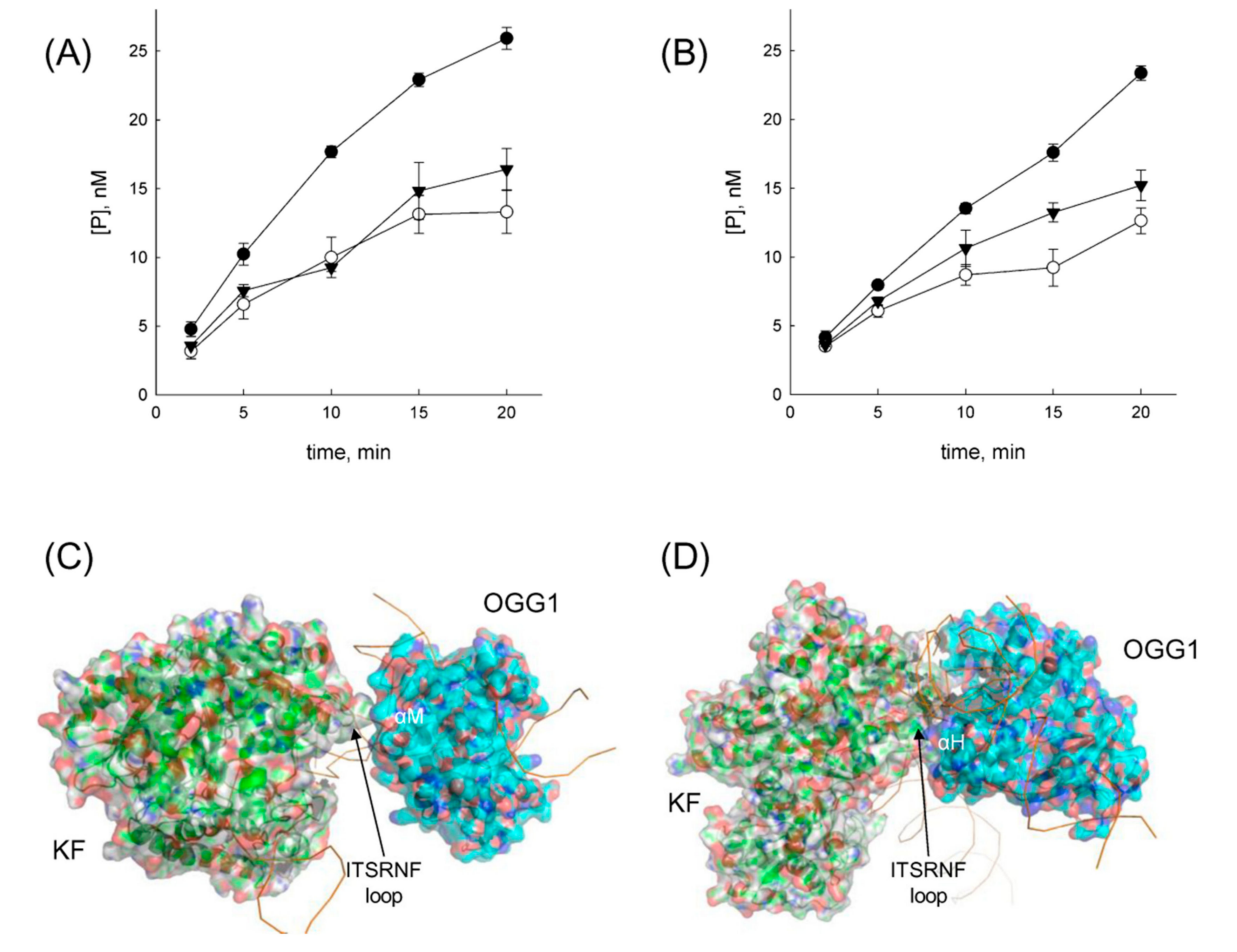
Displacement of OGG1 by Klenow fragment. (**A**) KF interacting with OGG1 bound to the template strand; (**B**) KF interacting with OGG1 bound to the downstream strand. White circles show the secondary substrate cleavage in the absence of KF, black circles, in the presence of KF and dNTPs and black triangles, in the presence of KF without dNTPs. Mean ± SEM of three independent experiments are presented; (**C**,**D**) possible structure of OGG1–KF encounter complex predicted by DNA-guided docking; OGG1 is on the downstream (**C**) or the template strand (**D**). OGG1 is colored cyan, KF, green, DNA is shown as a backbone trace.

We then inquired whether OGG1 can be displaced by a non-human DNA polymerase synthesizing a new DNA strand and unwinding the DNA helix ahead. Here we employed the Klenow fragment of *E. coli* DNA polymerase I (KF), an enzyme with strong strand displacement activity [50]. Since KF and OGG1 come from unrelated organisms, any OGG1 activity enhancement would most likely come from mechanical displacement rather than specific interactions with the DNA polymerase. An >2-fold enhancement of OGG1 turnover by KF was evident both when OGG1 was bound to either the template strand or the downstream strand (Figure 4A,B). Unlike POLβ, in the absence of dNTPs KF had no effect on OGG1 holding the template strand (Figure 4A), with some minor (not statistically significant) stimulation was observed with OGG1 on the downstream strand (Figure 4B). No difference in the cleavage of the secondary substrate alone was observed with or without KF (data not shown). When the primer was labeled, KF demonstrated efficient elongation (data not shown).

No structure of KF bound to the primer/template/downstream strand is available. However, KF from *Geobacillus stearothermophilus* in such a complex was recently studied by a combination of molecular dynamics and single-molecule fluorescence microscopy, and an all-atom model was generated, in which KF introduces a sharp kink into DNA and separates ~5 nucleotide pairs between the template and the downstream strand [38]. Using this model for DNA-guided docking between KF and OGG1 (Figure 4C,D), we have shown that OGG1, when present in the downstream strand, contacts the ^776^ITSRNF^781^ loop in the KF fingers subdomain upon the polymerase encounter through the same αM helix as was observed with POLβ. Interestingly, when OGG1 holds the template strand, the contact interface involves the same loop in KF, but the αI rather than αM helix in the glycosylase. The αI helix and the adjacent αH/αI make multiple contacts with the undamaged DNA strand opposite to the lesion, and their distortion may also be expected to dislodge OGG1 from its complex with DNA.

### 3.3. OGG1 Is Displaced by DNA Polymerases after Hydrolysis of a Schiff Base Intermediate

**Scheme 1:**
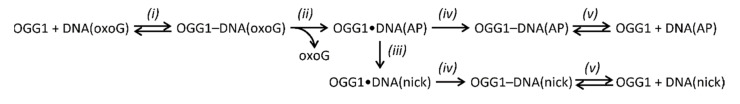
Reaction mechanism of OGG1. The shown steps are (**i**) damaged DNA-binding, (**ii**) oxoG release concerted with Schiff base formation, (**iii**) β-elimination, (**iv**) Schiff base hydrolysis, (**v**) DNA product release.

OGG1 is characterized by a multistep reaction mechanism. After the enzyme binds damaged DNA (Scheme 1i), the catalytic nucleophile Lys249 displaces the damaged base and forms a Schiff base covalent enzyme–DNA intermediate (Scheme 1ii). Part of the intermediate may undergo β-elimination, nicking DNA 3′ to the damaged site (Scheme 1iii). The Schiff base is then hydrolyzed (Scheme 1iv) and the abasic or nicked product, which has high affinity for the enzyme, is slowly released (Scheme 1v) [13,30,51]. The intermediate can be irreversibly trapped by reduction with NaBH_4_, and this reaction is often used to confirm that an active site amine rather than a water molecule acts as a nucleophile during base excision [52,53].

**Figure 5:**
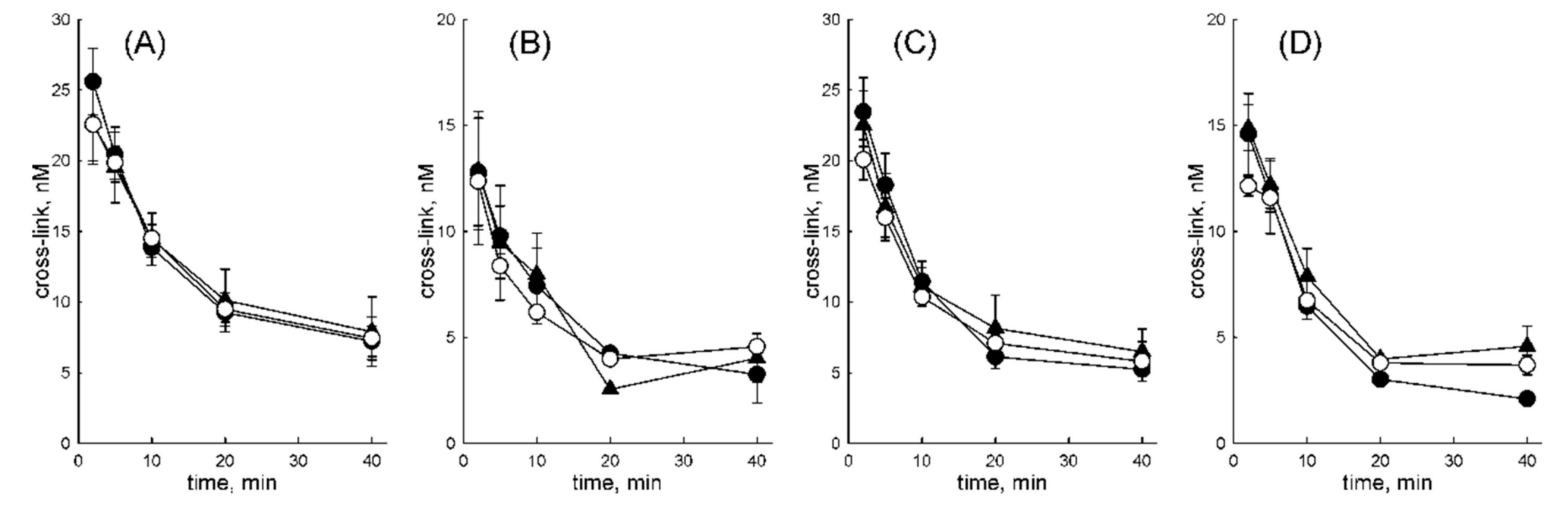
Time course of Schiff base decay in the reaction catalyzed by OGG1 in the presence and in the absence of DNA polymerases. (**A**) POLβ interacting with OGG1 bound to the template strand; (**B**) POLβ interacting with OGG1 bound to the downstream strand; (**C**) KF interacting with OGG1 bound to the template strand; (**D**) KF interacting with OGG1 bound to the downstream strand. White circles show the secondary substrate cleavage in the absence of a DNA polymerase, black circles, in the presence of a polymerase and dNTPs and black triangles, in the presence of a polymerase without dNTPs. Mean ± SEM of three independent experiments are presented.

When OGG1 is stimulated by AP endonuclease APEX1, the half-life of the covalent intermediate is significantly shortened, consistent with enforced displacement of OGG1 from the AP site product at an early reaction step [13,15]. We have inquired whether the observed displacement of OGG1 by DNA polymerases is also accompanied with the accelerated Schiff base hydrolysis (Scheme 1iv) or occurs at a later stage (Scheme 1v). When the fraction of the remaining NaBH_4_-trappable intermediate was measured in the absence of DNA polymerases, the half-life of 3.2–7.8 min^−1^ could be calculated (Figure 5) for OGG1 bound either to the template or the downstream strand in either POLβ or KF reaction buffers. The presence of DNA polymerases had no effect on the rate of the Schiff base decay (Figure 5). We conclude that both POLβ and KF can displace OGG1 from tight non-covalent complexes with the DNA product after Schiff base hydrolysis but are unable to disrupt the covalent OGG1–DNA conjugate even though it is labile and prone to spontaneous hydrolysis during the reaction.

### 3.4. OGG1 Can Be Displaced by a Nonspecifically Diffusing Protein

**Figure 6:**
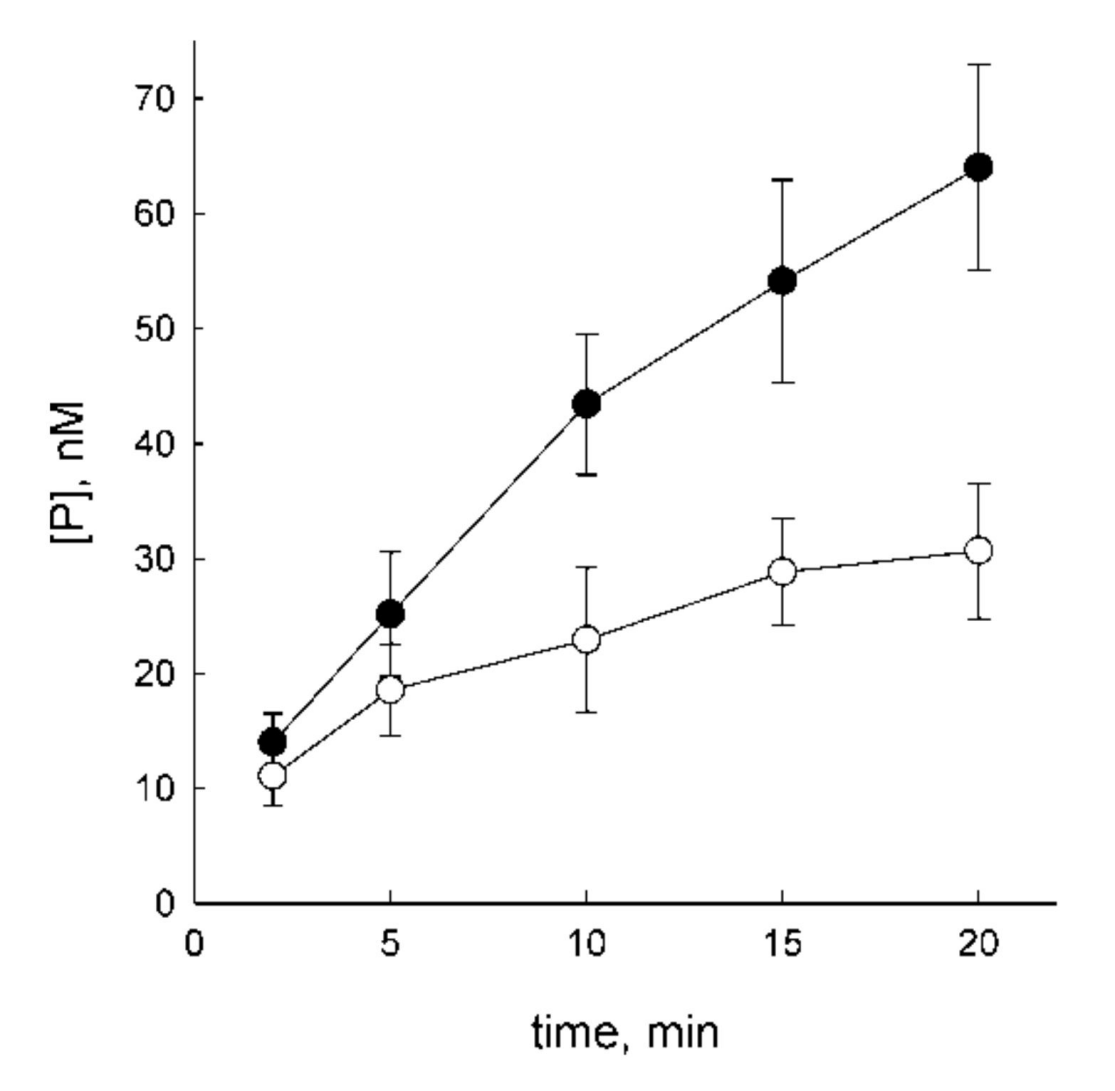
Displacement of OGG1 by D4 protein. White circles show the secondary substrate cleavage in the absence of D4, black circles, in the presence of D4. Mean ± SEM of three independent experiments are presented.

The apparent ability of POLβ to displace OGG1 in the absence of dNTPs raises the question whether other proteins capable of one-dimensional diffusion along DNA can do the same. To address this question, we have used the same experimental system with vaccinia virus D4 protein instead of a DNA polymerase. D4 forms part of the viral replication complex processivity subunit and, in addition, possesses uracil–DNA glycosylase enzymatic activity [54,55,56]. Free D4 protein forms a half-torus-shaped dimer [57] and can diffuse along DNA over ~250 nucleotides (E.A.D. and D.O.Z., paper in preparation). Despite being orthogonal to the human BER system, D4 was able to displace OGG1 from DNA with high efficiency; the secondary substrate cleavage was accelerated more twofold (Figure 6).

### 3.5. NEIL1 Can Be Displaced by Diffusing Proteins

NEIL1 is a bifunctional DNA glycosylase homologous to *E. coli* endonuclease VIII (Nei) [58]. After the removal of the damaged base, NEIL1, like OGG1, remains tightly bound to the AP reaction (*K*_d_ ~2–20 nM [59,60]), but the characteristic lifetime of this complex is 10–15 s [60], which is shorter than for OGG1. NEIL1 was reported to be stimulated in a similar manner to OGG1 by several DNA repair and replication proteins, including PCNA and 9–1–1 complexes [61,62], flap endonuclease 1 [63], Cockayne syndrome B protein [64], replication protein A [65], Werner helicase [66,67], replication factor C [68] and multifunctional regulatory YB-1 protein [69,70]. This multitude of interactions suggests that they may be nonspecific and related to protein–protein interaction mediated by one-dimensional diffusion.

**Figure 7:**
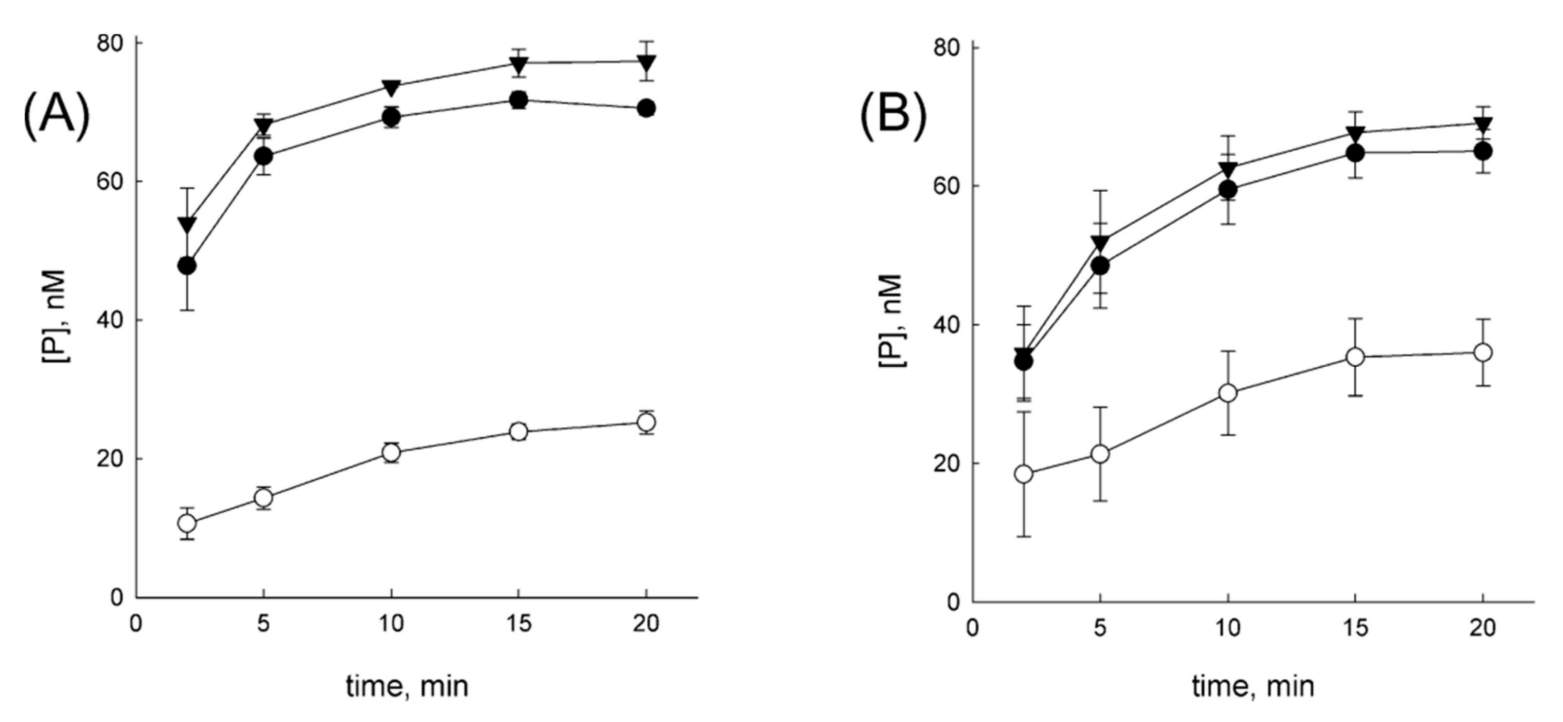
Displacement of NEIL1 by DNA polymerase β. (**A**) POLβ interacting with NEIL1 bound to the downstream strand; (**B**) POLβ interacting with NEIL1 bound to the template strand. White circles show the secondary substrate cleavage in the absence of POLβ, black circles, in the presence of POLβ and dNTPs and black triangles, in the presence of POLβ without dNTPs. Mean ± SEM of 3–4 independent experiments are presented.

Since NEIL1 is specific for oxidized pyrimidine bases and can also process AP sites [58], the experimental scheme remained the same, but the primary substrate contained an AP site, while the cleaved secondary substrate contained a 5-hydroxyuracil residue (ohU) [58]. POLβ was able to displace NEIL1 from the complex with the DNA reaction product with high efficiency, both when NEIL1 was in the template strand and in the downstream strand (Figure 7). The displacement was again equally efficient both with and without the polymerase reaction. D4 protein stimulated the cleavage of the secondary substrate to approximately the same extent as for OGG1 (data not shown). Thus, NEIL1 protein, which is less strongly bound to DNA, is easily displaced by other proteins even in the passive diffusion mode.

### 3.6. Cas9 Is a Barrier for Both Actively Moving and Passively Diffusing Proteins

*Streptococcus pyogenes* Cas9 RNA-guided DNA endonuclease makes a double-strand break and forms an extremely stable complex with the DNA product with the half-life > 40 h [71]. The reported *K*_d_ values for Cas9/RNA-binding to the DNA target are 0.5–2-nM [71,72]. Cas9 is bioorthogonal in human cells and most likely cannot specifically interact with their components. On the other hand, due to the wide use of Cas9 for genome editing, it is of interest how cell systems respond to it.

To assess the ability of DNA polymerases to displace such tightly bound, bulky obstacle (molecular weight ~160 kDa), we have modified our substrates and the reaction setup. The downstream sequence was re-designed to include the well-characterized Sp2 protospacer [33] and the protospacer-adjacent motif TGG. In addition, the length of the substrate was increased to ensure unhindered access of DNA polymerases to the primer 3′-terminus. In standard assays for Cas9 activity, the protein is taken in large excess over DNA because the exact amount of active enzyme in the reaction is hard to estimate due to the need for Cas9–sgRNA assembly [34]. In contrast, the displacement analysis requires all Cas9 molecules to be bound to the primary substrate. To optimize the reaction conditions, we tested several molar ratios of Cas9 to the primary substrate (0.2, 0.4, 0.6, 0.8, and 1.0). The ratio of 0.8 Cas9 molecules per one DNA molecule and a time interval of 2 to 60 min were chosen. Cas9 was first allowed to bind the RNA guide, and the assembled complex was incubated with an unlabeled primary substrate (P–DCas//TCas). The labeled secondary substrate (*S2Cas//Cas2S) was then added together with DNA polymerase, and its cleavage was followed.

**Figure 8:**
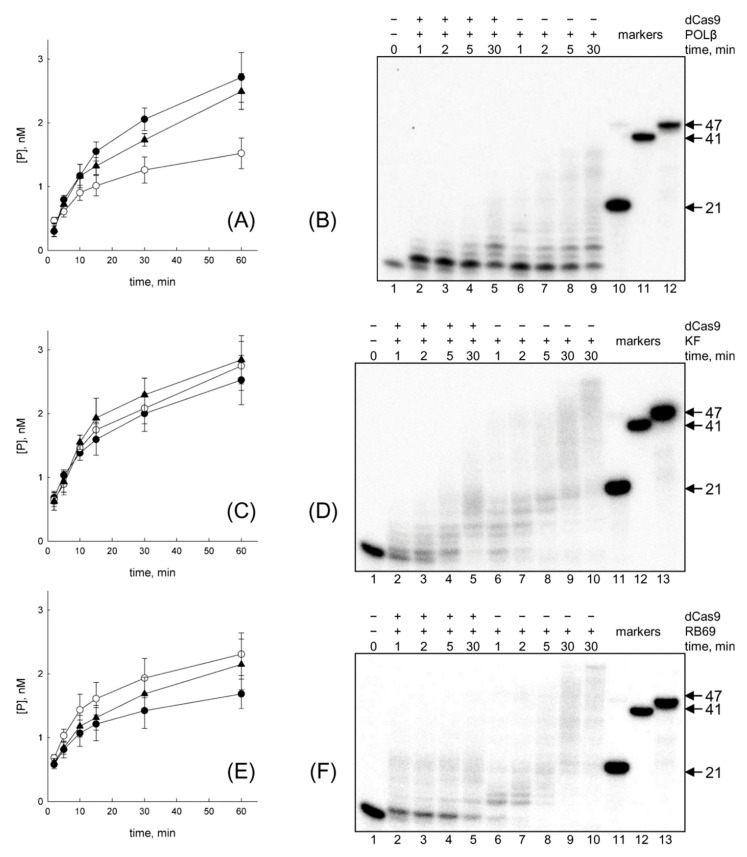
Displacement of Cas9 by DNA polymerases. (**A**) POLβ interacting with Cas9 bound to DNA; (**B**) primer extension by POLβ in the presence of dCas9; (**C**) KF interacting with Cas9 bound to DNA; (**D**) primer extension by KF in the presence of Cas9; (**E**) RB69 DNA polymerase interacting with Cas9 bound to DNA; (**F**) Primer extension by RB69 DNA polymerase in the presence of dCas9. In (**A**,**C**,**E**) white circles show the secondary substrate cleavage in the absence of a DNA polymerase, black circles, in the presence of a DNA polymerase and dNTPs and black triangles, in the presence of KF without dNTPs. Mean ± SEM of 3–5 independent experiments are presented. In (**B**,**D**,**F**) lane 1 is DNA substrate (primer/template/downstream strand, primer length 11 nt) without dCas9 or polymerase; lanes 2–5, DNA polymerase and dCas9 are present; lanes 6–9, only DNA polymerase is present; lane 10 in (**D**,**F**) DNA polymerase extending the primer/template construct without a downstream strand (absent from (**A**) since POLβ has very low activity without a downstream strand); lanes 11–13 (10–12 in (**A**)) mobility markers with their positions indicated by the arrows.

First, we inquired whether POLβ can displace Cas9. Although under the conditions of excess substrate the observed cleavage was low, consistent with the published data [71], the presence of POLβ led to a moderate, but statistically significant increase in product accumulation (Figure 8A). The observed stimulation suggests that, although human POLβ is unlikely to interact with *S. pyogenes* Cas9 specifically, it is able to displace tightly bound Cas9 from DNA in both elongation and passive diffusion mode. However, this required 10-fold more enzyme than the displacement of OGG1 and NEIL1 (100-nM vs. 10 nM).

We repeated the same experiments with KF and phage RB69 DNA polymerase, which also has strong strand displacement activity [73]. Unlike for POLβ, the presence of KF did not affect the efficiency of cleavage of the secondary substrate by Cas9 even at 100-nM polymerase (Figure 8C). The presence of RB69 DNA polymerase also did not increase the cleavage of the secondary substrate and even slightly decreased it (Figure 8E). The D4 protein was also unable to displace Cas9 from DNA (data not shown). Thus, the only protein capable of limited displacement of Cas9 was POLβ.

To test the effect of DNA-bound Cas9 on the ability of DNA polymerase to elongate the primer, an experiment was performed with the substrate *P–DCas//TCas, and the DNA synthesis with or without Cas9 was monitored. Since it was necessary to have an intact full-length DNA template to follow the passage of DNA-polymerases through the Cas9 footprint, we have used dCas9, a mutant variant of Cas9 (D10A H840A) devoid of the nuclease activity [33]. As in the experiments described above, the substrate was first incubated with dCas9 and sgRNA to form a tight complex, and then a DNA polymerase was added. Although POLβ, KF and RB69 can displace the downstream strand, primer elongation in the presence of the Cas9 obstacle proceeds was significantly hindered (Figure 8B,D,E). For POLβ both in the presence and in the absence of dCas9, a pronounced stop was observed after the incorporation of one and three nucleotides (Figure 8B), and further synthesis was appreciable only without dCas9. A comparison of the structures of a POLβ/primer/template/downstream strand complex (2FMP [37]) and a Cas9/DNA/sgRNA complex (5Y36 [74]) reveals that in our substrate, POLβ does not make contact with dCas9 even after the insertion of three nucleotides. This suggests that the inability of POLβ to conduct further synthesis is most likely due to its failure to displace the downstream strand stabilized by the interactions with dCas9.

KF and RB69 DNA polymerases have strong strand displacement activity and can distort covalently bound proteins to insert several nucleotides after the encounter of a protein–DNA cross-link [42]. Both polymerases were capable of synthesizing the full-length product in the absence of dCas9 but stopped at ~21 nt product length when dCas9 was bound to the downstream DNA part (Figure 8D,F). This corresponds to the maximal extension to ~1 nucleotide before the RNA/DNA heteroduplex within the Cas9 complex. No structure of RB69 polymerase bound to primer/template/downstream strand is available. However, a comparison of the KF model discussed above [38] and the Cas9/DNA/sgRNA structure [74] shows that the sharp kink at the template strand and partial melting of downstream DNA allow KF to approach the Cas9 molecule closely and stop very soon after the collision.

## 4. Discussion

Densely crowded conditions in the cell nucleus are well appreciated. Considering that many nuclear proteins have functions associated with nucleic acids-binding, a significant fraction of genomic DNA is covered by tightly associated structural, regulatory and catalytic proteins [4,6]. However, the competition between these proteins for DNA-binding and translocation along DNA was studied only sporadically.

Here, we have addressed two sides of this problem, centering on the functions of two classes of DNA-dependent proteins: DNA polymerases and DNA glycosylases. One particular aim was to see whether tightly bound proteins could impede progress of DNA polymerases. In a recent study, we demonstrated that when DNA polymerases encounter proteins covalently cross-linked to DNA, such obstacles are impassable even if located in a downstream strand normally displaced by the polymerase [42]. However, DNA polymerases varied in their ability to elongate the primer into the footprint of the cross-linked protein, some of them being able to proceed to the very point of the cross-link. This indicates that translocation of an elongating polymerase, which uses the energy of dNTP hydrolysis to move ahead [75,76], may generate enough force to distort the cross-linked protein globule. Would it be sufficient to displace a non-covalently bound protein? Our data suggest that it is possible for proteins bound to DNA with moderate affinity and low to moderate half-life (NEIL1 and OGG1), but strong and very long-living Cas9 complexes were mostly refractory to elongating DNA polymerases. The latter is consistent with the reports that catalytically dead Cas9 is blocking for replication and transcription in living cells [77,78]. It is quite probable that the affinity of proteins for DNA or the half-life of the protein–DNA complexes are key parameters determining their resistance to displacement by other proteins moving along DNA. In this case, proteins bound with ~1-nM affinity, as reported for Cas9 [71,72], would represent a prominent obstacle for the progress of other proteins, possibly requiring special means for their clearance. An extended range of tightly bound proteins and their competitors should be studied to arrive to a more quantitative picture of such displacement.

Quite unexpectedly, DNA polymerase β was able to displace bound OGG1 and NEIL1 even in the absence of dNTPs. Moreover, POLβ was the only polymerase that showed some displacement of Cas9, albeit at a higher (equimolar) polymerase:substrate ratio. Since DNA polymerases are known to use passive one-dimensional diffusion to slide along DNA in search of the primer end [79], we have inquired whether other sliding proteins can do the same. Vaccinia virus D4, a component of the processivity subunit of the viral replication complex, also displaced OGG1 and NEIL1, but not Cas9. OGG1 and many other DNA glycosylases are stimulated by APEX1, and stimulation of some DNA glycosylases including NEIL1 by a variety of DNA-binding proteins was reported (recently reviewed in [10]). Mechanistic studies of the stimulation revealed that APEX1 replaces OGG1 at the AP site product with the glycosylase remaining bound to DNA nearby; however, it is still debated whether active displacement occurs or APEX1 occupies the AP site briefly vacated by OGG1 [12,15,16]. In our experiment, the stimulation of OGG1 and NEIL1 by passively diffusing POLβ and D4 was nearly as efficient as the stimulation by elongating DNA polymerases, more consistent with the active displacement model.

Passively diffusing KF showed some tendency to stimulate OGG1, although it did not reach statistical significance. Although the affinities of KF and POLβ for nonspecific DNA were never compared directly under identical conditions, there are indications that their interactions with extended DNA duplexes may be structurally different. POLβ possesses an N-terminal 8-kDa domain, which is engaged in binding double-stranded DNA ahead of the elongating polymerase and has a 2′-deoxyribose-5′-phosphate activity important for BER. On undamaged double-stranded duplexes, this domain efficiently binds DNA and is required for one-dimensional diffusion of POLβ [80,81]. In contrast, DNA polymerase I holoenzyme interacts with the downstream DNA through its 34 kDa 5′→3′-exonuclease domain absent from KF [82,83]. Thus, it is possible that the presence of additional DNA polymerase domains specifically interacting with the downstream double-stranded DNA in the elongation complex increases the overall polymerase affinity for nonspecific DNA and allows it to compete with other DNA-binding proteins in the passive diffusion mode more efficiently.

Despite the pronounced ability of POLβ to displace obstacles in the passive diffusion mode, the mechanism of displacement is likely to be different from the polymerase in the elongation mode. The present model of POLβ searching for a nick or a gap in DNA involves non-specific binding of double-stranded DNA by the 8-kDa domain, diffusing over the distance of ~24 bp on average and probing for a primer 3′-end with the polymerase domain, with the whole polymerase molecule switching between open and closed conformations [80,81,84]. In the open conformation, the footprint of the eight-kilodalton domain is ~5 nt and fluorescence energy transfer measurements suggest that the catalytic domain extends by ~60 Å from the bound DNA, an equivalent of 18 base pair steps [80]. When the eight-kilodalton domain is bound to DNA in the diffusion mode, it can either interact itself with the protein obstacle or the obstacle may be contacted by the polymerase domain that, at the moment, is not engaged at the gap. The structure of searching POLβ is still lacking, making it difficult to make predictions about the interaction interfaces with OGG1 or other proteins. On the contrary, the elongation mode of POLβ has been extensively investigated, and the structure of POLβ bound to the gapped substrate is available. DNA-guided docking of POLβ and OGG1 complexes produced well-defined areas of possible contacts, amenable to further studies by molecular dynamics and site-directed mutagenesis. Notably, neither POLβ nor KF could disengage OGG1 from the covalent reaction intermediate but were only able to displace it after the Schiff base was hydrolyzed. Overall, the displacement of protein obstacles by elongating POLβ, KF and possibly other polymerases may be reminiscent to a “cowcatcher” model proposed for the disassembly of the RecA filament by the advancing DNA polymerase V during SOS-associated translesion DNA synthesis in *E. coli* [85,86], in which the strain put on the terminal monomer of the filament induces its ATP-independent release from DNA.

While BER stimulation through the displacement of slow-turnover DNA glycosylases by AP endonucleases is well characterized, much less is known about possible displacement of DNA glycosylases by other BER proteins. In a biologically relevant situation, POLβ may encounter OGG1 or NEIL1 during the repair of clustered lesions, a type of DNA damage often produced by ionizing radiation [87,88]. OGG1 efficiently processes oxoG near other base lesions, nicks and gaps in any DNA strand unless they are within 1–3 bases from the oxoG base [89,90] and POLβ filling the gap may collide with OGG1. In human cell extracts repairing tandem oxoG lesions (i.e., two oxoG bases in the same strand), the 5′-oxoG is nicked first, producing the situation in which POLβ could encounter OGG1 in the downstream strand [91]. However, a more important conclusion from our data are that not only POLβ, but also nonspecific proteins diffusing along DNA can collide with and displace DNA glycosylases from a complex with their product. This may be problematic for DNA integrity if an abasic site or a nick with an unprocessed 3′-end generated by the glycosylase is exposed rather than handed to APEX1. The tendency of DNA glycosylases to be displaced by molecular traffic may be one reason for the abundance of APEX1, which is present in a large excess over other BER proteins (10^1^–10^4^-fold); quantitative proteomics estimates suggest that the levels of APEX1 in the human nucleus is comparable with many structural nuclear proteins [92]. On the other hand, displacement of a DNA glycosylase by any nonspecifically diffusing protein could prevent the coordinated handover of the repair intermediate to APEX1 and shift BER from its main branch to some minor subpathways. For example, BER can proceed in an APEX1-independent manner, with the AP site removed by the AP lyase function of NEIL1 (and possibly its homologs NEIL2 and NEIL3) and the 3′-end at the nick processed by polynucleotide kinase/3′-phosphatase PNKP1 [93]. Moreover, human cells possess APEX2, an APEX1 homolog that has low AP endonuclease activity, but efficiently removes blocking deoxyribose derivatives, such as β-elimination products, from the 3′-end [94].

Generally, our results are consistent with the view of molecular traffic on DNA as a factor that can displace and stimulate DNA glycosylases and thus accelerate the first step of BER. However, it is less clear whether the overall BER fidelity suffers as the result of this uncoordinated glycosylase displacement. In the cell, BER is orchestrated by the XRCC1 scaffold protein, which was shown to interact with APEX1 [95], POLβ [96,97,98], DNA ligase IIIα [99,100,101], OGG1 [102] and NEIL1 [93]. However, rather than delivering a full complement of BER enzymes to the damaged site as a single complex, XRCC1 is believed to facilitate the exchange of the sequentially acting BER enzymes as the lesion is processed [103,104]. It is possible that even if a glycosylase is displaced by POLβ or a nonspecifically wandering protein, XRCC1-dependent APEX1 loading would still prevent the unwanted consequences of a nonprotected AP site.

## Figures and Tables

**Table 1:** Structures of the oligonucleotides used in this work.

ID ^1^	Sequence (5′→3′)	Length	Modification
P	CGAGACCGTCG	11	
DC	GAGGAAAGAAGCGAAGGAATTCCAGAGC	28	
DG	GAGGAAAGAAGGGAAGGAATTCCAGAGC	28	
DU	GAGGAAAGAAGUGAAGGAATTCCAGAGC	28	dU
DX	GAGGAAAGAAGXGAAGGAATTCCAGAGC	28	X = oxoG
DCas	GAGGAAACTGATAACTCAATTTGTAAAAAATGGTACTG-AGC	41	
TC	GCTCTGGAATTCCTTCCCTTCTTTCCTCTCGACGGTCTCG	40	
TG	GCTCTGGAATTCCTTCGCTTCTTTCCTCTCGACGGTCTCG	40	
TU	GCTCTGGAATTCCTTCUCTTCTTTCCTCTCGACGGTCTCG	40	dU
TX	GCTCTGGAATTCCTTCXCTTCTTTCCTCTCGACGGTCTCG	40	X = oxoG
TCas	GCTCAGTACCATTTTTTACAAATTGAGTTATCAGTTTCC-TCTCGACGGTCTCG	53	
S2X	CTCTCCCTTCXCTCCTTTCCTCT	23	X = oxoG
S2Y	CTCTCCCTTCYCTCCTTTCCTCT	23	Y = ohU
2S	AGAGGAAAGGAGCGAAGGGAGAG	23	
S2Cas	CTGATAACTCAATTTGTAAAAAATGGTACTGAGCA	35	
Cas2S	TGCTCACTACCATTTTTTACAAATTGAGTTATCAG	35	
M21	CGAGACCGTCGAGAGGAAACT	21	
M41	CGAGACCGTCGAGAGGAAACTGATAACTCAATTTGTAA-AAA	41	
M47	CGAGACCGTCGAGAGGAAACTGATAACTCAATTTGTAA-AAAATGGTA	47	

^1^ In the text, the substrates are designated as P–D//T or P//T, where P is the primer, D is one of the downstream strand oligonucleotides, T is one of the template oligonucleotides. If necessary, the labeled strand is marked with an asterisk, e.g., * P//T.
